# LSPR based on-chip detection of dengue NS1 antigen in whole blood

**DOI:** 10.1039/d1ra05009e

**Published:** 2021-10-15

**Authors:** S. Lathika, A. Raj, A. K. Sen

**Affiliations:** Fluid Systems Lab, Department of Mechanical Engineering, Indian Institute of Technology Madras Chennai India ashis@iitm.ac.in; Micro Nano Bio Fluidics Group, Indian Institute of Technology Madras Chennai India; Department of Mechanical Engineering, Indian Institute of Technology Patna Patna India

## Abstract

The development of a biosensor for rapid and quantitative detection of the dengue virus continues to remain a challenge. We report a lab-on-chip device that combines membrane-based blood plasma separation and a localized surface plasmon resonance (LSPR) based biosensor for on-chip detection of dengue NS1 antigen from a few drops of blood. The LSPR effect is realized by irradiating UV-NIR light having a spectral peak at 655 nm onto nanostructures fabricated *via* thermal annealing of a thin metal film. We study the effect of the resulting metal nanostructures on the LSPR performance in terms of sensitivity and limit of detection, by annealing silver films at temperatures ranging from 100 to 500 °C. The effect of annealing temperature on the nanostructure size and uniformity and the resulting optical characteristics are investigated. Further, the binding between non-targeted blood plasma proteins and NS1-antibody-functionalized nanostructures on the LSPR performance is studied by considering different blocking mechanisms. Using a nanostructure annealed at 200 °C and 2X-phosphate buffer saline with 0.05% Tween-20 as the blocking buffer, from 10 μL of whole blood, the device can detect NS1 antigen at a concentration as low as 0.047 μg mL^−1^ within 30 min. Finally, we demonstrate the detection of NS1 in the blood samples of dengue-infected patients and validate our results with those obtained from the gold-standard ELISA test.

## Abstract 2

A lab-on-chip device that combines membrane-based blood plasma separation and a localized surface plasmon resonance (LSPR) based biosensor for on-chip detection of dengue NS1 antigen from a few drops of blood.

## Graphical abstract

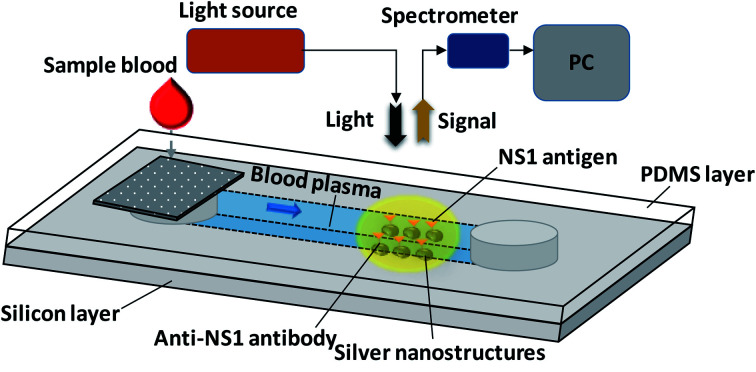

## 1. Introduction

Dengue is a vector-borne viral disease caused by the dengue virus (DENV) which infects hundreds of millions of people every year in more than a hundred countries.^1^ According to the world health organization (WHO), the number of dengue cases is on the rise, posing a global threat.^2,3^ Despite its increased transmission rate in recent years, an effective antiviral therapy against dengue is still in its infancy, and therefore, early diagnosis is the only solution.^4^ The single-positive stranded RNA virus, which belongs to the family Flaviviridae, secretes the dengue nonstructural glycoprotein (NS1) antigens during the early phase of infection. Different laboratory-based diagnostic tests are available, enzyme-linked immunosorbent assay (ELISA) being one of the most reliable serological tests for the detection of dengue virus.^5^ ELISA still has its limitations such as prolonged testing time (∼6 h), labor intensiveness, the requirement of multiple reagents, and numerous washing steps.^6^ In recent decades, attempts have been extensively made to overcome these limitations and develop point-of-care diagnostic devices for rapid and accurate tests for the dengue virus. Rapid diagnostic tests (RDTs)^7^ utilizing immunochromatographic principles require low sample volumes and provide results in <90 min, but they are less accurate and sensitive than ELISA. To achieve detection speed comparable to that of RDTs without compromising on the accuracy and sensitivity, microfluidics-based biosensors have been developed for dengue detection.^8–10^ The inherent limitations of electrical and electrochemical sensing mechanisms^8,9^ in terms of pH dependency and reproducibility are overcome by optical techniques.^10^ Recently, highly sensitive optical detection techniques such as localized surface plasmon resonance (LSPR)^11^ have been developed for dengue detection.

Plasmonic metal nanoparticles such as gold and silver have drawn significant attention from researchers in recent decades due to their unique optical, electromagnetic, chemical, and biological properties.^12–14^ Extensive study of silver nanoparticles have been carried out owing to their unique optical properties, that can be utilized to develop nanoscale optical, chemical, and biological sensors.^12^ FTIR spectrum of silver nanoparticles has shown a strong interaction between different functional groups and silver surfaces.^13^ Similarly, gold nanoparticles are also popular for biosensing applications owing to their ease of preparation, biocompatibility, and inertness.^14^ Even though gold nanoparticles are preferred due to their biocompatibility and chemical stability, silver nanoparticles offer better sensitivity and a broad absorption spectrum (300–1200 nm).^15^ The wide absorption spectrum is attributed to the LSPR phenomenon, arising from the excitation of electrons in the conduction band, which oscillate coherently when irradiated with light.^16^ The LSPR phenomena are characterized by the change in the dielectric properties of the environment around the nanoparticles.^17–19^ Red-shift in absorption dips observed in LSPR with silver nanospheroids on TiO_2_ surface owing to the refractive index change was used for the detection of selective binding of biotin and streptavidin.^17^ Theoretical studies have shown that for thin analyte layers, the sensitivity of the LSPR to the surrounding refractive index change is comparable with a traditional surface plasmon resonance sensor.^18^ Further, LSPR-based label-free biosensing devices typically rely on the change in the local refractive index and predominantly all organic bio-molecules have higher refractive indices than buffer.^19^ Based on the above principles, LSPR based biosensor devices have been developed for the rapid detection of various analytes.^20,21^ Also, LSPR with gold nanoparticles on a thin film has been used for highly sensitive detection of other biochemical entities, such as human osteoblast cells.^22^ A comprehensive review of plasmonic-based biosensors used in viral diagnostics has been reported recently.^23^

Despite a significant development of the field, two important questions remain unanswered, which we attempt to address in the current work. First, the size and uniformity of the nanoparticles that affect LSPR performance have not received much attention. Second, the possible binding between non-targeted plasma proteins with the antibody-functionalized surface, and protocols to minimize such cross interactions is not well understood. Nanostructures created using thermal annealing can have varying particle size, interparticle spacing, and uniformity, as the particles are formed due to hydrodynamic instability which is sensitive to the operating temperature. Similarly, nanostructures grown on a surface from the spin coating of nanoparticle suspensions can agglomerate and/or get distributed randomly resulting in similar variations. Therefore, it is important to understand the effect of the physical characteristic of the nanostructures on the optical behavior and consequently the LSPR performance. Further, lab-on-chip based diagnostic devices typically detect dengue NS1 antigens from blood plasma sample which contains the numerous other proteins, which are either simple proteins or conjugated proteins. Moreover, the average concentration of the plasma proteins, which is typically between 6–8% in healthy individuals, can vary in diseased conditions.^24^ The variation in the average plasma protein concentrations among individuals along the serological cross-reactivity between closely related other arboviruses makes diagnosis very challenging.^25^ Therefore, it is important to control the binding of non-targeted proteins present in the plasma other than the target NS1 protein with the antibody functionalized nanoparticle surfaces to remove the chances of false positivity/negativity and improve specificity.

Here, we study the effect of the variation of nanoparticle size and uniformity which is achieved by varying the annealing temperature, on the LSPR performance in terms of sensitivity and limit of detection. We also investigate the binding between non-targeted blood-plasma proteins with NS1-antibody-functionalized nanostructures on the LSPR performance by considering different blocking mechanisms. Based on the above studies, we develop an improved lab-on-chip device that combines membrane-based blood plasma separation and a biosensor based on localized surface plasmon resonance (LSPR) for on-chip detection of dengue NS1 antigen from a few drops of blood. Finally, the detection of dengue NS1 antigens in the blood samples of dengue infected patients is demonstrated and our results are compared with that obtained from the conventional ELISA test.

## 2. Description of the device and detection assay

**Fig. 1:**
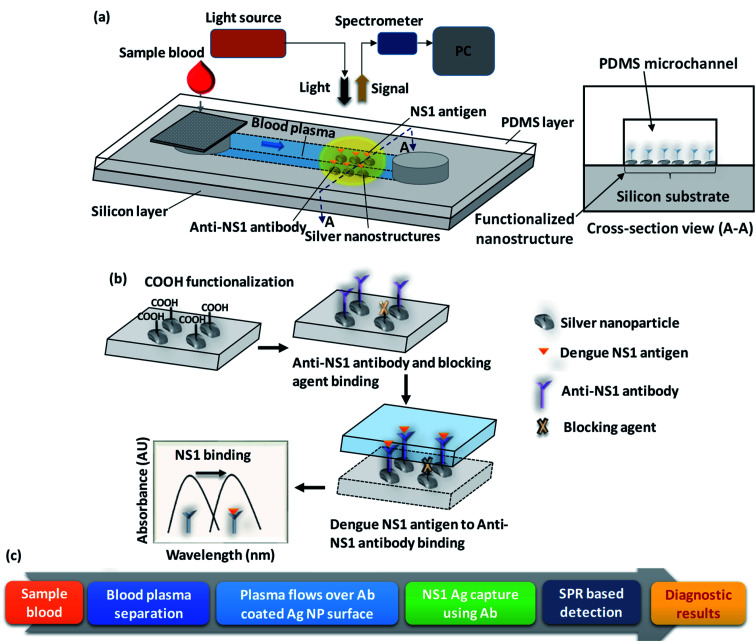
(a) Schematic of experimental set-up for detection of localised surface plasmon resonance (LSPR) based dengue NS1 antigen, (b) process steps showing surface functionalization, antibody/antigen binding, and wavelength-shift, (c) a flow chart of the operating protocols from sample introduction to test results.

A schematic of the lab-on-chip device used for the rapid detection of dengue NS1 antigen from whole blood is presented in Fig. 1a. The device comprises a two-layered structure: the top layer is a polydimethylsiloxane (PDMS) microchannel of 1000 μm width and 100 μm height and the bottom later is a silver-nanostructured silicon substrate functionalized with anti-NS1 antibody. A cross-sectional view of the device at the functionalized nanostructure region is shown in Fig. 1a. The detailed procedure used for fabricating the integrated device is presented in the experimental section. Drops of sample blood are introduced into the device at the device inlet which houses a membrane filter to separate the plasma into the microchannel by filtering out blood cells. The blood plasma runs through the microchannel due to capillary flow and contacts the anti-NS1 antibody functionalized region. A schematic of the functionalization steps and a flow chart of the working of the device is depicted in Fig. 1b and c. First, the nanostructure is functionalized with reactive carboxyl (–COOH) groups and then the anti-NS1 antibody is allowed to bind to the COOH functionalized nanoparticles. The –COOH functionalized nanoparticles unbound with the anti-NS1 antibody are then encapsulated with a blocking agent to prevent binding of NS1 antigen and prevent false-positive signal.

When the plasma sample comes in contact with the anti-NS1 antibody functionalized region, the dengue NS1 antigens bind to the anti-NS1 antibody, which changes the refractive index of the region surrounding the nanoparticles. Since the sizes of the nanoparticles are smaller than the wavelength of light, the irradiation of the nanoparticles to light gives rise to the localized surface plasmon resonance (LSPR) phenomenon, which is a result of the interactions between the incident light and surface electrons in a conduction band.^26^ The LSPR signal is captured using a suitable photodetector. As the plasmon resonant frequency is highly sensitive to the refractive index of the surrounding a change in refractive index arising from the change in the surface molecules due to antibody–antigen binding results in a shift in the resonant frequency, consequently the wavelength corresponding to a maximum LSPR signal. The shift in the resonant frequency and therefore the corresponding wavelength is proportional to the refractive index change so it is higher when a higher concentration of NS1 is present in the plasma sample. Herein, we study the effect of the nanoparticle size and uniformity and the binding of non-targeted plasma proteins with the blocking agent on the LSPR performance.

## 3. Experimental

### 3.1 Materials

Silicon wafer is procured from Semiconductor Technology and Applications, USA. Acetone and isopropyl alcohol are purchased from Fisher Scientific, USA. Methanol is purchased from Finar, India. Trichloroethylene, nitric acid, hydrofluoric acid (HF), 3-aminopropyltriethoxysilane (APTES), 11-mercapto undecanoic acid (MUA), 3-mercapto propionic acid (MPA), *N*-(3-dimethylaminopropyl)-*N*′-ethyl carbodiimide hydrochloride (EDC), *N*-hydroxy succinimide (NHS), and phosphate buffered saline (PBS) are purchased from Sigma-Aldrich, USA. Sylgard 184 elastomer and curing agent are purchased from Dow Corning, USA. Polyethersulfone membrane (PSM0180-A) is supplied by Cobetter Filtration, China.

### 3.2 Methods

#### 3.2.1 Fabrication of nanostructured silver substrate

100 mm P-type silicon substrate is used for silver nanostructure fabrication. The substrate is immersed consecutively in trichloroethylene, acetone, and nitric acid, boiling at 180 °C for 4 min. Subsequently, it is immersed in 1 : 10 deionized (DI) diluted hydrofluoric acid (HF) for 30 s. The substrate is deposited with a 5 nm thick silver film *via* electron beam evaporation (BOC Edwards Auto 306, UK) at 10^−6^ Torr. Each substrate is then annealed separately inside a furnace at 100 °C, 200 °C, 300 °C, 400 °C, and 500 °C for 1 h. Finally, the constructed substrate is allowed to cool down to room temperature, and diced manually into rectangular substrates of desired dimension (0.75 cm × 1 cm) and used. The effect of the resulting nanostructures with different annealing temperatures on the LSPR performances is studied.

#### 3.2.2 Surface functionalization of nanostructures

**Fig. 2:**
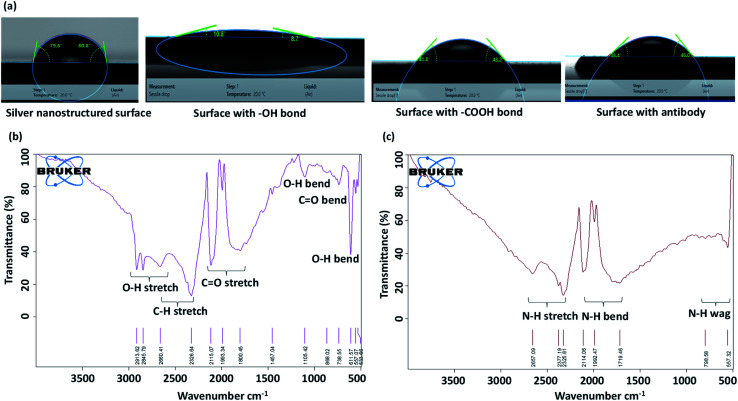
(a) Water contact angles of the original silver nanostructured surface, and the surfaces after each surface treatment step, (b) FTIR measurement results for the surface after functionalization with COOH group, (c) FTIR measurement results for the surface after binding with anti-NS1 antibodies.

Initially, the annealed substrate is ultrasonicated with 5 mL of acetone, methanol, and isopropanol for 5 min each consecutively. Then the substrate is immersed in boiling acetone at 56 °C for 5 min. First, the cleaned substrate is treated in an oxygen plasma chamber at ∼400 mTorr and 10 W for 5 min for the creation of hydroxyl (–OH) groups on the surface. Next, 50 μL of 2% solution of 3-aminopropyltriethoxysilane (APTES) prepared in ethanol is added to the plasma-treated substrate for 90 min that leads to the formation of the amine group. Then, the substrates are washed with ethanol for removal of any excess APTES and then treated with 50 μL of a mixture of 20 mM 11-mercapto undecanoic acid (MUA) and 3-mercapto propionic acid (MPA) at a ratio of 1 : 9 overnight at room temperature. Further, the substrate is washed with ethanol and the carboxylic (COOH) group created in the previous step are activated by adding 50 μL of a mixture of 0.15 M *N*-(3-dimethylaminopropyl)-*N*′-ethyl carbodiimide hydrochloride (EDC) and 0.03 M *N*-hydroxy succinimide (NHS) at a ratio of 1 : 1 for 1 h at room temperature. Finally, the substrate is washed with phosphate buffer saline (PBS) to remove any traces of EDC and NHS. This functionalization step aims to create –COOH terminal end which covalently binds with the amine group of antibodies. The water contact angles of the original silver nanostructured surface, and the surfaces after each surface treatment step is measured using a goniometer (DSA25 KRUSS GmbH) and the corresponding images are presented in Fig. 2a. We see that the silver nanostructured surface, which is initially near-hydrophobic becomes hydrophilic with the creation of hydroxyl and carboxyl groups on the surface that facilitates binding of the surface with antibodies. Further, Fourier-transform infrared spectroscopy (FTIR) measurements are performed which show the creation of carboxyl groups on the surface, as shown in Fig. 2b.

#### 3.2.3 Integration of functionalized nanostructure and PDMS microchannel

Anti-NS1 antibody (Abcam, USA) is diluted with phosphate-buffered saline (PBS) at a ratio of 1 : 10. The diced functionalized substrate (0.75 cm × 1 cm) was coated with 50 μL of diluted anti-NS1 antibody and incubated at 4 °C for 3 h. Then, the substrate was rinsed with PBS to remove unbound antibodies. To prevent the binding of NS1 antigen with the free carboxylic group of NHS, 50 μL of 100 μg mL^−1^ BSA with 0.5% Tween-20 surfactant was added and incubated at 4 °C overnight. FTIR measurement is performed to show the creation of amine groups on the surface after anti-body binding, as shown in Fig. 2c. The substrate was washed with PBS and then bonded with a layer of PDMS channel of width 1000 μm and height 100 μm having a cylindrical inlet of 4 mm diameter. The PDMS channel was fabricated by using standard photolithography and soft lithography process which is detailed elsewhere.^27^ Since PDMS is normally hydrophobic, to enable capillary flow, the channel is made hydrophilic by treating it in a plasma chamber (Harrick Plasma, USA) for 2 min at 30 W power. The hydrophilicity of the PDMS channel can be retained for several weeks by dipping the channel in deionized water.^27^ A 340 μm thick polyethersulfone membrane consisting of 1.8 μm pores was cut into 5 mm × 5 mm size, moistened with 35 μL of heparin for 15 s and fixed over the channel inlet (Fig. 1a).

#### 3.2.4 Detection of dengue NS1 antigen spiked in the whole blood sample of healthy individuals

The dengue NS1 antigen is spiked in whole blood received from healthy donors at varying concentrations ranging from 0.25 μg mL^−1^ to 2.0 μg mL^−1^. Further, the NS1 antigen in the spiked blood is detected by using the method described as follows. A drop of a whole blood sample of 10 μL volume spiked with NS1 antigen at a particular concentration is placed on the polyethersulfone membrane above the inlet. The membrane separates the plasma into the channel inlet which flows through the channel *via* capillary action. The device is then incubated at 4 °C for 30 min. Next, the PDMS channel layer is peeled off from the substrate, and the substrate is washed with PBS buffer, and then the LSPR measurements are performed to measure the absorption characteristics. For each NS1 concentration, the experiments were repeated at least three times, and the mean and standard deviation are calculated.

#### 3.2.5 Detection of NS1 antigen in dengue infected patients

All experiments were performed in accordance with the “Indian Council of Medical Research (ICMR) National Ethical Guidelines for Biomedical Research involving Human Participants”, and approved by the ethics committee at the “Indian Institute of technology Madras”. Informed consents were obtained from human participants of this study. The blood samples of dengue patients were collected from the IIT Madras institute hospital in vacutainers with 7.2 mg K2 containing ethylene diaminetetraacetic acid (EDTA) (BD, New Jersey, USA) and the experiments were performed within 30 min of sample collection. The sample was also tested at the hospital laboratory using the conventional ELISA test (Euroimmun NS1 kit).

#### 3.2.6 Non-specific binding of plasma proteins

The binding of non-targeted bio-molecules to the anti-NS1 antibody is demonstrated without spiking blood samples with NS1 antigen. Experiments are performed with both whole and deproteinized plasma on substrates annealed at 200 °C. For deproteinization of plasma, a spin column vial (QIAGEN, Germany) is used to filter out proteins that are >10 kDa. After the functionalization step, the nanostructured surface is immobilized with a 1 : 10 diluted anti-NS1 antibody at 4 °C for 3 h. The anti-NS1 antibody is diluted in PBS prepared by dissolving one PBS tablet (Sigma-Aldrich, USA) in 200 mL of deionized water, which gives 0.01 M PBS with a pH of 7.4. After PBS wash, the unbound surface of the substrate was encapsulated using various blocking agents overnight at 4 °C as mentioned below: (i) 3% and 5% Bovine Serum Albumin (BSA) in PBS, (ii) 1% and 3% BSA in 0.01 M PBS (iii) 1% and 3% BSA in 0.01 M PBS with 0.05% Tween-20 surfactant (iv) 0.01 M and 0.02 M PBS wash buffer with 0.05% Tween-20 surfactant. The blocking agents were prepared by mixing the various reagents at the specified ratios with the help of a vortex shaker. After removal of the unbound blocking agent, two such substrates are incubated with whole and deproteinized plasma, in parallel. The procedure is repeated with different plasma samples acquired from different healthy adults. LSPR signal is captured after each wash step for each substrate and the comparison between the signals obtained with whole and deproteinized plasma is evaluated to determine the extent of non-specific binding.

### 3.3 LSPR experimental setup

The system consists of a deuterium-halogen light source (DH-2000-BAL, Ocean Optics, Germany), an optical fibre coupler based reflection probe (QR-400-7-UV-VIS, Ocean Optics, Germany), a miniature spectrometer (Flame-T, Ocean Optics, Germany), and XYZ-translation stages (Thor Labs, USA) (Fig. 1a). Light from the source, coupled with optic fibre, propagates through the coupler and irradiates onto the substrate. The reflected light then returns to the coupler and finally propagates to the spectrophotometer. The acquired LSPR based absorbance spectrum is analysed using OceanView software (Ocean Optics, Germany) by setting up the parameters such as integration time, the number of scans, and boxcar width to 15 ms, 25, and 50 respectively, and corrected for nonlinearity errors. The distance between the reflection probe and biosensor device is adjusted to 9.0 mm which gives a beam diameter of 4.5 mm.

## 4. Results and discussion

### 4.1 Effects of annealing temperature on silver nanostructure – physical and optical characteristics

**Fig. 3:**
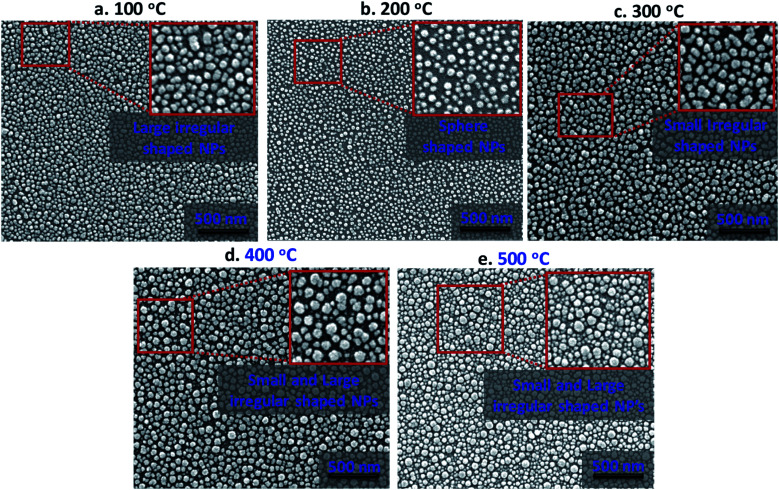
High resolution scanning electron microscopic images (HRSEM) of silver nanostructures annealed at (a) 100 °C, (b) 200 °C, (c) 300 °C, (d) 400 °C, and (e) 500 °C. The insets in the images show closeup views of the shapes of the nanoparticles (NPs) at the corresponding temperatures.

The absorption characteristics of silver nanostructures annealed for 1 h at different annealing temperatures in the range of 100–500 °C inside a furnace are studied in terms of morphological evolution, inter-particle separation distance, and polydispersity index. Fig. 3 shows high-resolution scanning electron microscope (HRSEM) images of the annealed silver nanostructures. The morphological evolution of silver nanoparticles (AgNPs) depends primarily on the thickness of the deposited silver film, annealing duration, and annealing temperature. Here, the thickness of the deposited silver film and annealing duration is kept fixed as 5 nm and 1 h respectively, as a film thickness of 5 nm is found to offer maximum absorption, lower scattering, and good areal coverage,^28^ and the effect of annealing temperature on the silver nanostructure is studied. The variation in the annealing temperature resulted in the formation of the distinct configurations of AgNPs such as isolated sphere-shaped NPs, larger and smaller irregular-shaped NPs (see insets in Fig. 3a–e). The annealed silver nanoparticles are of near-spherical shape with variations in particle size (*d*) in the range 21–48 nm and inter-particle distance (*δ*) in the range 28–56 nm.

The overall morphological evolution of AgNPs primarily depends on the temperature-dependent surface diffusion, sublimation, and surface energy minimization mechanism.^29,30^ The formation of three-dimensional (3-D) nanostructures can be explained in terms of the Volmer–Weber growth model which says, silver adatoms can diffuse and bind together on the surface with an adequate amount of thermal energy. Therefore, the annealing temperature has direct control over the surface diffusion coefficient (*D*_s_) and, diffusion length (*L*_d_) of adatoms, which is given by the relation 
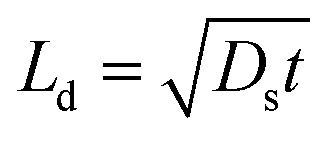
 where *t* is the residence time of adatoms on the surface, and *D*_s_ = *D*_o_ exp(−*E*/*KT*), where *E*, *T*, and *K* is energy barrier for surface diffusion, growth temperature, and Boltzmann constant, respectively. Considering the above relations, *L*_d_ is directly proportional to the temperature and surface diffusion is enhanced with the increase in temperature resulting in more compact binding of nanoparticles.^31^ Here, the binding energy between the Ag adatoms is stronger than the binding energy between the Ag and substrate atoms. In addition, the AgNPs change their size and configuration with an increase in annealing temperature in order to minimize their surface energy.^30^ The distribution of the nanoparticles is characterized in terms of polydispersity index (PDI), which is defined as the ratio of the standard deviation of the particle size (*s*_d_) to the average particle size (*d̄*), PDI = (*s*_d_/*d̄*).

The size of the nanoparticles has a significant impact on their optical characteristics. Further, the physicochemical properties of a nanomaterial act very differently from those of its bulk counterpart, primarily because of its enhanced surface interactions and quantum confinement effect. The particles in the nanoscale regime exhibit quantum confinement of electrons, *i.e.* quantum confinement effect. When a photon of energy equal to or greater than the bandgap energy hits a nanoparticle, electrons excite from valence band to conduction band. In the case of smaller NPs, only fewer atoms are required to create the particles and therefore the smaller NPs have a larger bandgap. Consequently, more energy is required to excite the electrons that ultimately results in a blue-shift in the case of smaller NPs and a red-shift for larger NPs.^32^ The above effect can be explained by the equation *E* = *hc*/*λ*_m_, where *E* is the energy, *h* is the Planck's constant, *c* is the speed of light and *λ*_m_ the wavelength of the absorption maxima.^33^ Similarly, the interdistance between the NPs can also affect the LSPR performance. When the centre-to-centre interparticle distance *δ* ≤ 2.5*d*, dipole–dipole interaction occurs due to small interparticle distance which affects the near-field coupling effect or resonance condition in the longitudinal direction.^34^

**Fig. 4:**
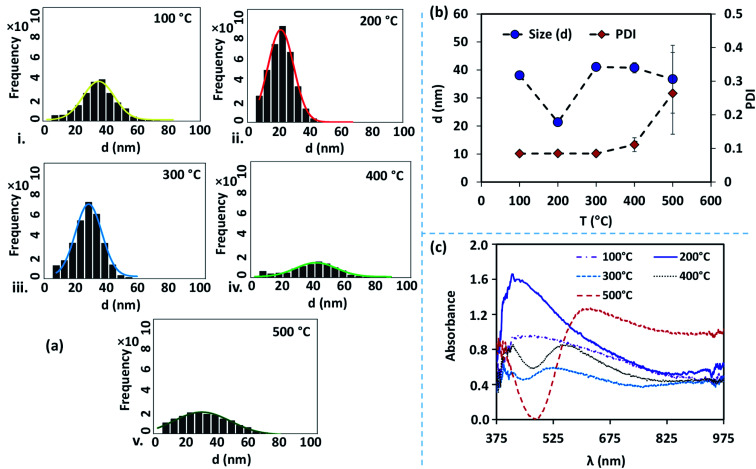
(a) Histograms showing the particle size of silver nanostructures annealed at five different temperatures: (i) 100 °C, (ii) 200 °C, (iii) 300 °C, (iv) 400 °C, v. 500 °C. (b) Particle size and polydispersity index (PDI) with annealing temperature (*T*) in °C. (c) Absorbance spectra of nanostructures at different annealing temperatures. Absorption is normalized with the peak absorption observed at the annealing temperature of 100 °C.

The size distribution of the AgNPs annealed at different temperatures is presented using particle size-frequency histograms (see Fig. 4a (i–v)). This data is used to obtain the variation of the NP size, and PDI *versus* the annealing temperature (see Fig. 4b). The error bars in experimental data are calculated by performing at least five independent measurements at the same experimental condition. It is observed that the AgNPs annealed at 100 °C are found to have an average particle size of 38 ± 1.5 nm, and PDI = 0.084, and the inter-particle distance is found to be 28.48 ± 5.68 nm. A relatively lower annealing temperature leads to the formation of irregular AgNPs, and limited surface diffusion of Ag adatoms giving rise to a higher PDI. At 100 °C, the separation of the AgNPs is limited and the temperature is just adequate to initiate the surface diffusion, resulting in a smaller interdistance between the particles. When the annealing temperature is increased to 200 °C, the surface diffusion is enhanced resulting in a more compact binding of nanoparticles forming isolated and spherical AgNPs (see Fig. 3b). The average size of the nanoparticles annealed at 200 °C is 21 ± 0.36 nm with a prominent interparticle distance of 33.74 ± 5.02 nm and a much smaller PDI = 0.085. Both at 100 °C and 200 °C, the relatively smaller size of the AgNPs offers a higher bandgap and thus gives rise to a blue-shift in the peak absorbance wavelength (see Fig. 4c). As the size of the AgNPs is relatively smaller at 200 °C compared to that at 100 °C, the peak wavelength *λ*_m_ = 415 nm is found to be smaller at 200 °C compared to that *λ*_m_ = 474 nm at 100 °C.

At annealing temperatures >200 °C, both the average size of the AgNPs and PDI increase with an increase in the annealing temperature. At 300 °C, 400 °C and 500 °C, the average sizes of the AgNPs are measured to be 41.09 ± 0.40 nm, 40.8 ± 1.85 nm, and 36.72 ± 12.13 nm, respectively, the interparticle distance are measured to be 43.11 ± 7.78 nm, 56.57 ± 9.14 nm, and 48.5 ± 12.04 nm, respectively, and the PDI are calculated to be 0.085 nm, 0.111 nm, and 0.264 nm, respectively. With an increase in the annealing temperature, the growth of the AgNPs is accelerated due to the coalescence and repeated recrystallization, in order to minimize surface energy resulting in larger size particles. The larger size of the AgNPs offer a smaller bandgap and therefore result in the occurrence of a red-shift in the peak absorbance wavelength (see Fig. 4c). As the average size of the AgNPs increases with the annealing temperature, the peak wavelength at 300 °C is *λ*_m_ = 526 nm, which is found to be smaller than *λ*_m_ = 549 nm at 400 °C, which is further smaller compared to *λ*_m_ = 623 nm at 500 °C. The variation of the peak absorbance wavelength *λ*_m_ with annealing temperature is also seen from Fig. 4c. Annealing the surfaces below 100 °C does not result in the formation of nanoparticles and above 500 °C, repeated agitation, coalescence, and crystallization resulted in the formation of much larger and smaller-sized nanoparticles with leading to a very high polydispersity, and a large standard deviation observed. Further, we observe that the peaks of the LSPR absorption band in case of 300 °C and 400 °C are split, which could be attributed to the large and inhomogeneous particle distribution seen at these two temperatures in Fig. 3. The existence of a bimodal LSPR peak has also been reported in the literature for silver nanoparticles.^35^

### 4.2 LSPR performance of the device at various annealing temperatures

**Fig. 5:**
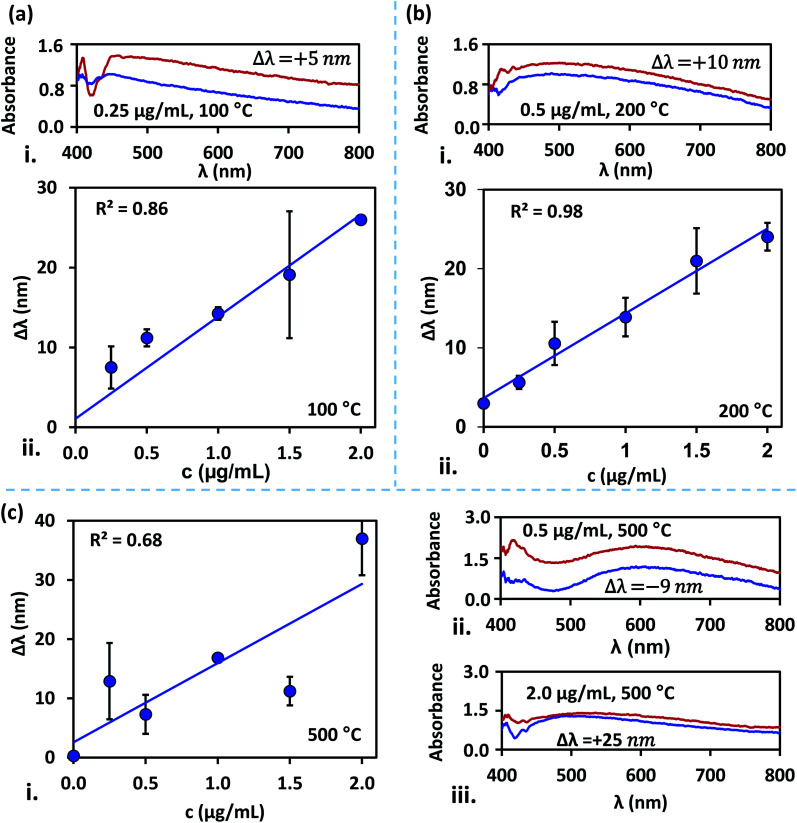
Variation of wavelength shift (Δ*λ*) with concentration (*c*) of NS1 antigen and variation of absorbance with wavelength (*λ*) using nanostructured surfaces fabricated at different annealing temperatures: (a) (i) Δ*λ versus c* at 100 °C, (ii) absorbance *versus λ* at 100 °C and *c* = 0.25 μg mL^−1^, (b) (i) Δ*λ versus c* at 200 °C, (ii) absorbance *versus λ* at 200 °C and *c* = 0.5 μg mL^−1^, (c) (i) Δ*λ versus c* at 500 °C, (ii) absorbance *versus λ* at 500 °C and *c* = 0.5 μg mL^−1^, (iii) absorbance *versus λ* at 500 °C and *c* = 2.0 μg mL^−1^, absorption is normalized with the peak absorption observed for the antibody functionalized surface.

The difference in the peak absorbance wavelength between the LSPR signals obtained before and after the dengue NS1 antigens binding with the anti-NS1 antibody-coated surfaces, Δ*λ* is measured. Measurement of Δ*λ* is performed for blood samples spiked with NS1 antigen at different concentrations in the range 0.25–2 μg mL^−1^ for the silver nanostructured surfaces annealed at different temperatures in the range 100–500 °C, and the results are presented in Fig. 5. It is found that irrespective of the annealing temperature of the substrates, a linear variation of Δ*λ* with NS1 antigen concentration is observed. Regression analysis of silver film annealed at 200 °C exhibits the best linear fit with *R*^2^ = 0.98, wherein the nanostructures are monodisperse with a much smaller PDI = 0.085 and spheroidal in shape, and interparticle distance ranging from tens to hundreds of nanometres. The results for substrates annealed at temperatures 100 °C and 300 °C exhibits moderate linear-fit with *R*^2^ values of 0.86 and 0.84, respectively, which is attributed to the fact that at these temperatures the silver nanostructures are comparatively larger, have deficit spheroidal shape and non-uniform inter-structural spacing. The large standard deviations observed at 1.5 μg mL^−1^ at 100 °C can possibly be attributed to surface contamination or improper washing.

The size of the silver nanostructures increased with an increase in annealing temperature to 400 °C and 500 °C due to particle–particle coalescence and the particles across the substrate are non-uniform in size which corresponds to the poor linear-fit. The nanostructures can affect the shift in the wavelength at peak absorbance. The surface plasmon peak is seen to shift towards red throughout NS1 concentration of 0.25–2 μg mL^−1^ for samples annealed at 100 °C and 200 °C (Fig. 5a and 4b). However, annealing at higher temperatures has contributed to both red and blue shifts (Fig. 5c). The contradictory observations of red and blue shifts are attributed to a higher polydispersity index (Hou D. *et al.*, 2002). For substrates annealed at 200 °C, we observed a limit of detection (LOD) of 0.047 μg mL^−1^ with a sensitivity of 10.72 nm (μg mL) for the detection of NS1 dengue antigen, and the correlation between wavelength shift (Δ*λ*_m_) and NS1 concentration (*c*) is as follows, Δ*λ*_m_ = 3.637 + 10.715*c*. Here, LOD is determined using, LOD = (3 × standard deviation of the control)/the slope of the linear fit.^36^

### 4.3 Non-specific binding of plasma proteins – blocking agents and LSPR performance

**Fig. 6:**
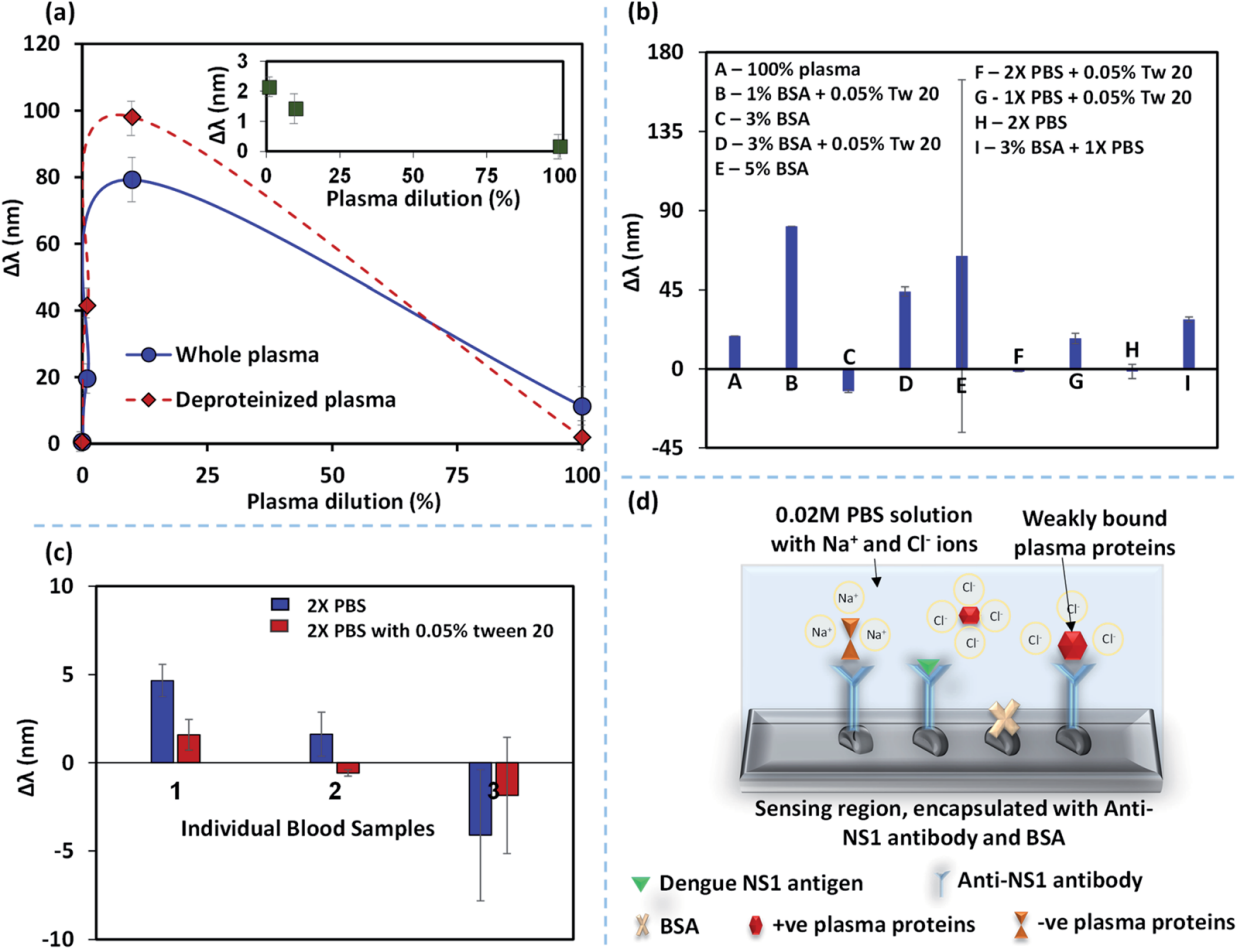
(a) The difference between the wavelength-shift (Δ*λ*) in case of whole plasma and deproteinized plasma at different dilution (%), inset shows the contribution of the smaller proteins <10 kDa towards the wavelength shift, (b) effect of the different blocking mechanisms on the non-specific binding represented in terms of Δ*λ*, (c) comparison of blocking performance of 2X buffer and 2X buffer with 0.05% Tween for different blood samples, (d) a schematic illustrating the blocking mechanism enabled by PBS with 0.05% Tween.

When a blood sample without spiked NS1 antigen was introduced into the device, we observed a considerable shift in the wavelength corresponding to peak absorbance intensity. This shift in the wavelength is attributed to the binding of non-specific plasma proteins with the anti-NS1 functionalized NPs that can lead to false-positive signals.^37–39^ Here, we study the non-specific binding of plasma proteins by performing experiments with whole blood plasma and deproteinized plasma at different dilutions. The deproteinized plasma is obtained by centrifuging the whole blood plasma in a spin column vial to filter out proteins >10 kDa, as discussed in the experimental section. So the deproteinized plasma contains proteins <10 kDa. The difference between the wavelength shifts in the case of whole blood and deproteinized plasma at different dilutions is shown in Fig. 6a and the contribution of the small proteins <10 kDa on the overall wavelength shift in the whole plasma is shown in the inset. It is observed that the effect of the presence of smaller proteins is less than 20% which indicates that the shift in the wavelength is mainly caused by the larger proteins >80%. Removal of larger proteins using a spin column, although reduces the non-specific binding of proteins, it does not eliminate the non-specific binding. Further, the use of spin-column introduces additional process steps, costs, and complexity into the protocol and therefore is not amenable to the lab-on-chip-based point of care applications. We also observe that the non-specific binding is negligible at higher dilutions and the difference between the whole and deproteinized plasma decreases with an increase in the dilution. Therefore, dilution of blood plasma is a simpler solution to address non-specific binding but this will reduce the concentration of NS1 in a blood sample and affect the limit of detection and sensitivity of the assay. Therefore, we explore alternative methods to address the problem of non-specific binding. Bovine serum albumin (BSA), an inert-carrier protein agent that interacts minimally with other proteins and also escorts low-solubility proteins at *in vivo* conditions. The surfactant Tween-20, which is added to PBS washing buffer in ELISA, has proven to be an excellent washing buffer to reduce the non-specific binding.^40^

The wavelength shifts obtained for different analytes: (i) BSA (1%, 3% and 5%), (ii) 1% and 3% BSA + 0.05% Tween-20, (iii) 0.01 M and 0.02 M PBS with 0.05% Tween-20, are presented in Fig. 6b. Although BSA was used as a blocking agent, it is found that BSA can also lead to binding with some of the plasma proteins^41^ and therefore we see distinct plasmonic shifts at different concentrations of BSA. The large standard deviation observed for 5% BSA could have arisen due to stochastic interaction and binding between BSA and plasma proteins. It is found that 0.02 M PBS buffer, with or without 0.05% Tween-20 results in the minimal plasmonic wavelength shifts. However, experiments with different blood samples showed a minimal non-specific binding for 0.02 M PBS buffer, with 0.05% Tween-20 (Fig. 6c). PBS has Na^+^ and Cl^−^ ions and these ions cluster around the weakly bound plasma protein to anti-NS1 antibody complex and partially neutralizes the charges and prevent the blood plasma proteins from binding with the anti-NS1 antibody as shown in Fig. 6d. Tween-20 is a non-ionic surfactant generally added to buffers which reduces the non-specific proteins by blocking the unreacted sites.^42^ The highly concentrated PBS along with 0.05% Tween-20 has an increased amount of Na^+^ and Cl^−^ ions that can shield or exchange charges with non-specific proteins.

### 4.4 Detection of dengue NS1 antigen in blood samples of dengue-infected patients

**Fig. 7:**
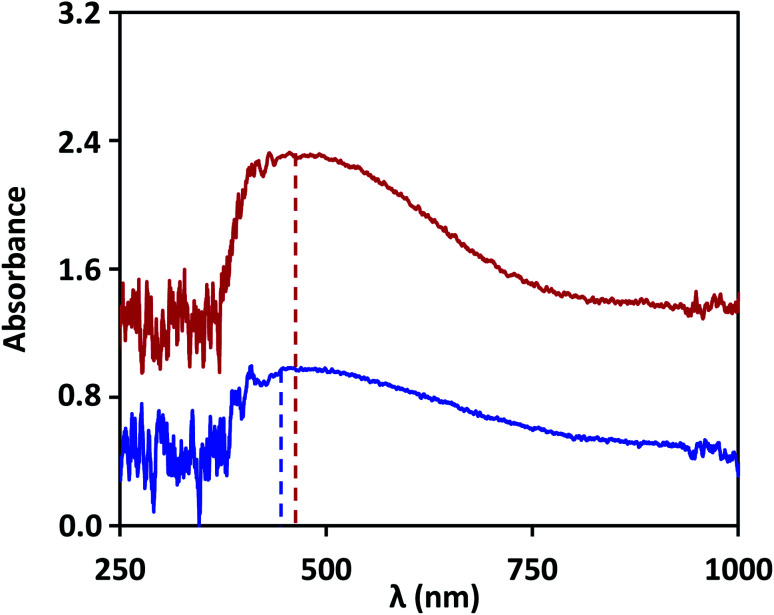
The wavelength shifts of absorption spectra after incubation of 200 °C annealed silver nanostructures with anti-NS1 antibody (blue) and dengue-infected patient samples (red). Absorption is normalized with the peak absorption observed for the antibody functionalized surface.

The lab on chip device is used to demonstrate detection of NS1 in the blood samples of dengue infected patients and the results are validated with that obtained using ELISA test. Based on our study, the device used silver nanostructures fabricated at an annealing temperature of 200 °C, and the non-specific binding was prevented by blocking the unbound nanostructures with 2X-PBS + 0.5% Tween-20. Dengue patient samples were obtained using the protocol described in the experimental section. LSPR signal was captured for the anti-NS1 antibody-coated surface and then a few drops (total 10 μL) of patient blood sample is introduced into the device. After the NS1 antigen in the sample binds to the antibody functionalized surface, the LSPR signal shows a shift in wavelength, as shown in Fig. 7. The wavelength shift is found to be 10 ± 2 nm, and by using the linear correlations developed in the present study, we find this corresponds to an NS1 concentration of 0.5 μg mL^−1^, indicating the samples are dengue positive. Literature reports that the clinical level varies between 0.04 to 2 μg mL^−1^ during the acute phase of dengue infection.^43^ The blood samples were further tested using the traditional laboratory technique, ELISA (Euroimmun NS1 kit). The patient samples were tested positive when analysed using the commercial kit, which has a calibrated scale as follows: test result data <8 indicates negative, >8 to <11 indicates borderline and ≥11 indicates positive. For the samples of the patient, the test data was found to yield a value of 11 RU mL^−1^, which indicates that the test is positive. Our study suggests that the lab on a chip device can be used for quantitative detection of dengue NS1 antigen from the blood sample of dengue infected patients.

## 5. Conclusions

We presented a lab-on-chip biosensor device that combines blood plasma filtration and localized surface plasmon resonance for on-chip detection of dengue NS1 antigen from sample blood. We studied the effect of silver nanostructures fabricated using annealing at different temperatures in the range of 100–500 °C on the LSPR performance in terms of measurement accuracy and sensitivity and limit of detection. The results showed that nanostructures annealed at 200 °C results in a more uniform nanostructure and optimal size range that results in a sharp peak in the absorbance spectrum. Detection of dengue NS1 antigen using substrates annealed at 200 °C resulted in a consistent variation in wavelength shift *versus* NS1 concentrations with a higher slope of the curve and higher correlation index of the linear-fit. The binding of nonspecific blood-plasma proteins with nanostructures that lead to false-positive signals was studied by considering different blocking mechanisms. It is found that 2X-phosphate buffer saline with 0.05% Tween-20 could significantly suppress the possibility of nonspecific binding. With the surface annealed at 200 C and 2X-phosphate buffer saline with 0.05% Tween-20 as the blocking agent, detection of dengue NS1 antigen at a concentration as low as 0.047 μg mL^−1^ could be performed within 30 min. The detection of NS1 antigen in the blood samples of dengue infected patients was demonstrated and the results were validated with the ELISA test.

## Ethical statement

All methods were carried out following relevant guidelines and regulations. All experimental protocols were approved by the Institute Ethics Committee, Indian Institute of Technology Madras (Ref. No. IEC/2020/02/AK-1/01). Informed consent was obtained from all subjects or if subjects are under 18, from a parent and/or legal guardian.

## Conflicts of interest

There are no conflicts to declare.

## Supplementary Material
